# Genetics of renal cancer: focus on MTOR

**DOI:** 10.18632/aging.100937

**Published:** 2016-03-26

**Authors:** Arindam P. Ghosh, Sunil Sudarshan

**Affiliations:** Department of Urology, University of Alabama at Birmingham, Birmingham, AL 35294, USA

**Keywords:** mTOR, rapamycin, renal cancer, mutations

Renal cell carcinoma (RCC) has multiple subtypes and may occur in hereditary and sporadic forms. Sporadic renal cell carcinomas are most commonly clear cell cancers (80%). Metastatic disease is found at presentation in almost 30% of patients with renal cell carcinoma and treatment of RCC metastases is greatly different from the treatment regimens of the primary tumor. Currently, several FDA approved therapies exist for metastatic clear cell RCC (ccRCC) which includes two rapamycin analogs-everolimus and temsirolimus. The mammalian target of rapamycin (mTOR) is a serine/threonine kinase and catalytic subunit of two biochemically distinct complexes called mTORC1 and mTORC2. Recently published TCGA data report aberrations in the PI3K/AKT/mTOR pathway in up to 28% of RCC cases [1]. Whether these aberrations predict for clinical benefit of mTOR-targeted therapy in ccRCC patients is debatable. Prior studies have identified hyperactivating point mutations in mTOR that remain sensitive to rapamycin [2] while other recent studies have identified a somatic mutation in mTOR that is resistant to allosteric mTOR inhibition while remaining sensitive to mTOR kinase inhibitors [3]. Mutations in *MTOR* are clustered in various regulatory domains in ccRCC. We focused our attention on a prominent cluster of hyperactivating mutations in the FAT (FRAP–ATM–TTRAP) domain of mTOR in ccRCC that led to an increase in both mTORC1 and mTORC2 activities and led to an increased proliferation of cells [4]. Several of the FAT domain mutants demonstrated a decreased binding of the intrinsic inhibitor DEPTOR (DEP domain containing mTOR-interacting protein), while a subset of these mutations showed altered binding of the negative regulator PRAS40 (proline rich AKT substrate 40). We also identified a recurrent mutation in *RHEB* (Ras homolog enriched in brain) in ccRCC patients that exclusively increased mTORC1 activity. Interestingly, mutations in the FAT domain of *MTOR* and in *RHEB* remained sensitive to rapamycin, though several of these mutations demonstrated residual mTOR kinase activity after treatment with rapamycin at clinically relevant doses. Overall, our data suggests that point mutations in the mTOR pathway may lead to downstream mTOR hyperactivation through multiple different mechanisms to confer a proliferative advantage to a tumor cell.

Given the central role of mTORC1 as a downstream target of PI3K activity, there exists a clear rationale for targeting mTORC1 in cancer and using rapalogs clinically. Unfortunately, the effectiveness of rapamycin as a single agent therapy is fraught with several limitations. mTORC1 promotes IRS-1 degradation [5] implying that the potential therapeutic benefit of inhibiting mTORC1 with rapamycin is opposed by the release of feedback inhibition of PI3K/AKT activation. In addition to inhibition of the feedback loop that restrains PI3K/AKT activation, everolimus treatment in breast cancer patients can increase ERK activation by a mechanism which is largely unknown, thereby adding a new level of complexity to allosteric inhibition of mTORC1 by rapalogs [5]. In an attempt to target the mTOR pathway more effectively, novel ATP competitive inhibitors that act at its catalytically active site are being developed. Active-site inhibitors have indeed proved more effective inhibitors of cell proliferation than rapamycin in a variety of tumor subtypes *in vitro* as they have a distinct advantage in that they inhibit 4E-BP1 phosphorylation at rapamycin resistant sites and also block AKT phosphorylation at Ser473 [6]. Significant homology in the kinase domains of PI3K and mTOR has made possible the development of dual active-site inhibitors. While these agents can circumvent the activation of PI3K/AKT feedback loops activated by rapalogs, dual PI3K/mTOR inhibitors could lead to activation of alternative compensatory pathways. The elucidation of the feedback loops that regulate the outputs of signaling networks is an area of fundamental importance for the rationale design of effective anticancer drugs that can be used in conjunction with PI3K/AKT/mTOR inhibitors.

While most of mTOR targeted therapies including everolimus and temsirolimus target mTORC1, mTORC2 is emerging as a pivotal player in many cancers. Defining mTORC2's role in the cellular milieu has been more challenging compared to mTORC1 because of its insensitivity to acute rapamycin treatment. As mTORC2 is a key regulator of cell proliferation and metabolic reprogramming of tumor cells [7], there is an increasing need to design therapeutic agents that specifically target mTORC2. Our *in vitro* data suggests that these hyperactivating mutations confer relative resistance to rapalog therapy and these findings may have dosing implications for patients with ccRCC. These findings may be highly relevant from a clinical point of view, as *MTOR* mutations could serve as biomarker predicting tumor responses to mTOR allosteric inhibitors and explain acquired resistance to this class of drugs in humans.
